# Supplementary figures and images for: Tracking clonal and plasmid transmission in colistin- and carbapenem-resistant Klebsiella pneumoniae
Source: mSystems. 2025 Jan 10;10(2):e01128-24. doi: 10.1128/msystems.01128-24 (PMC11834398; doi:10.1128/msystems.01128-24)

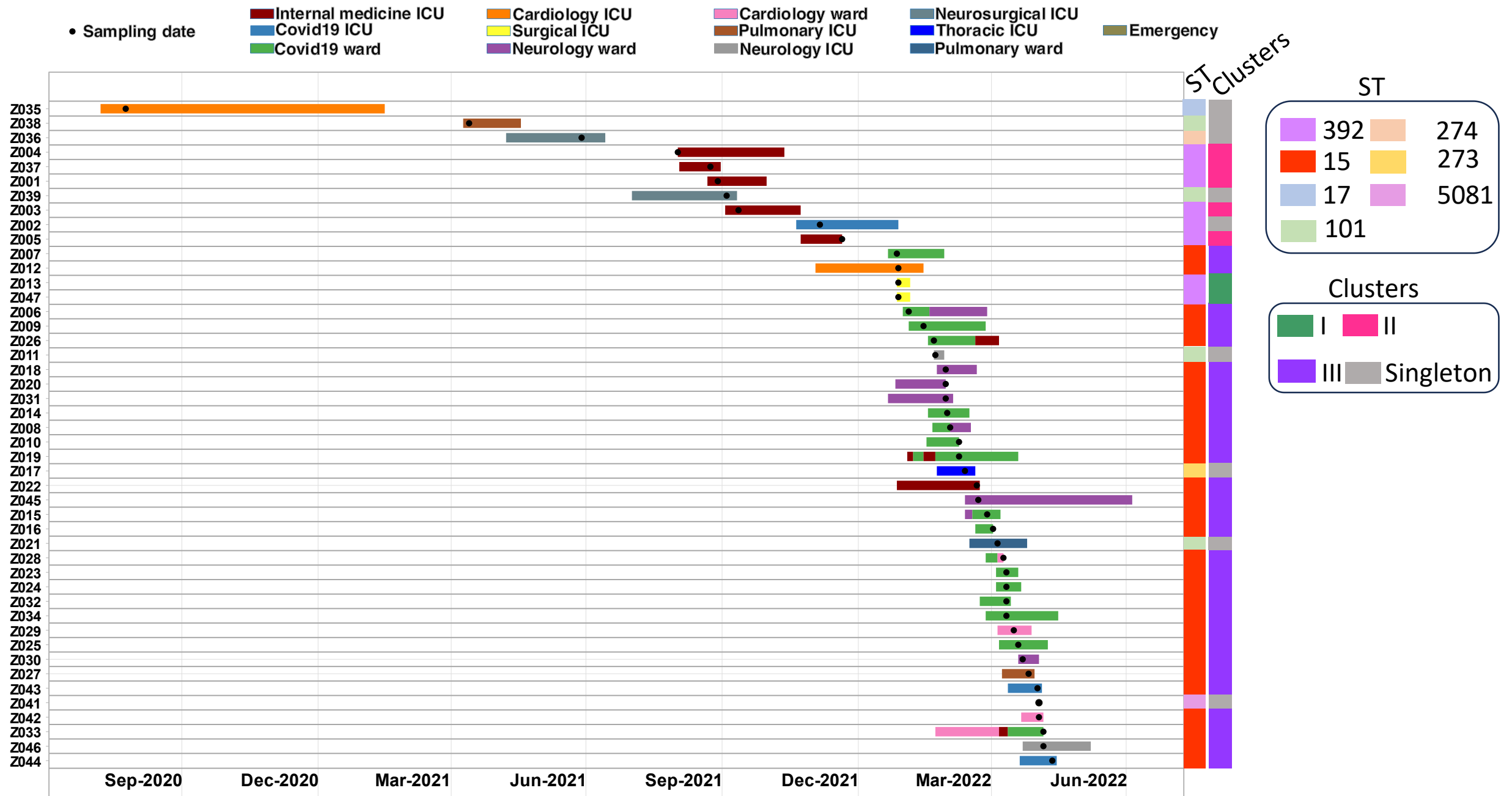

Supplement: Fig. S1 — Spatial and temporal relationship of all patients within the hospital. [file msystems.01128-24-s0001.pdf]

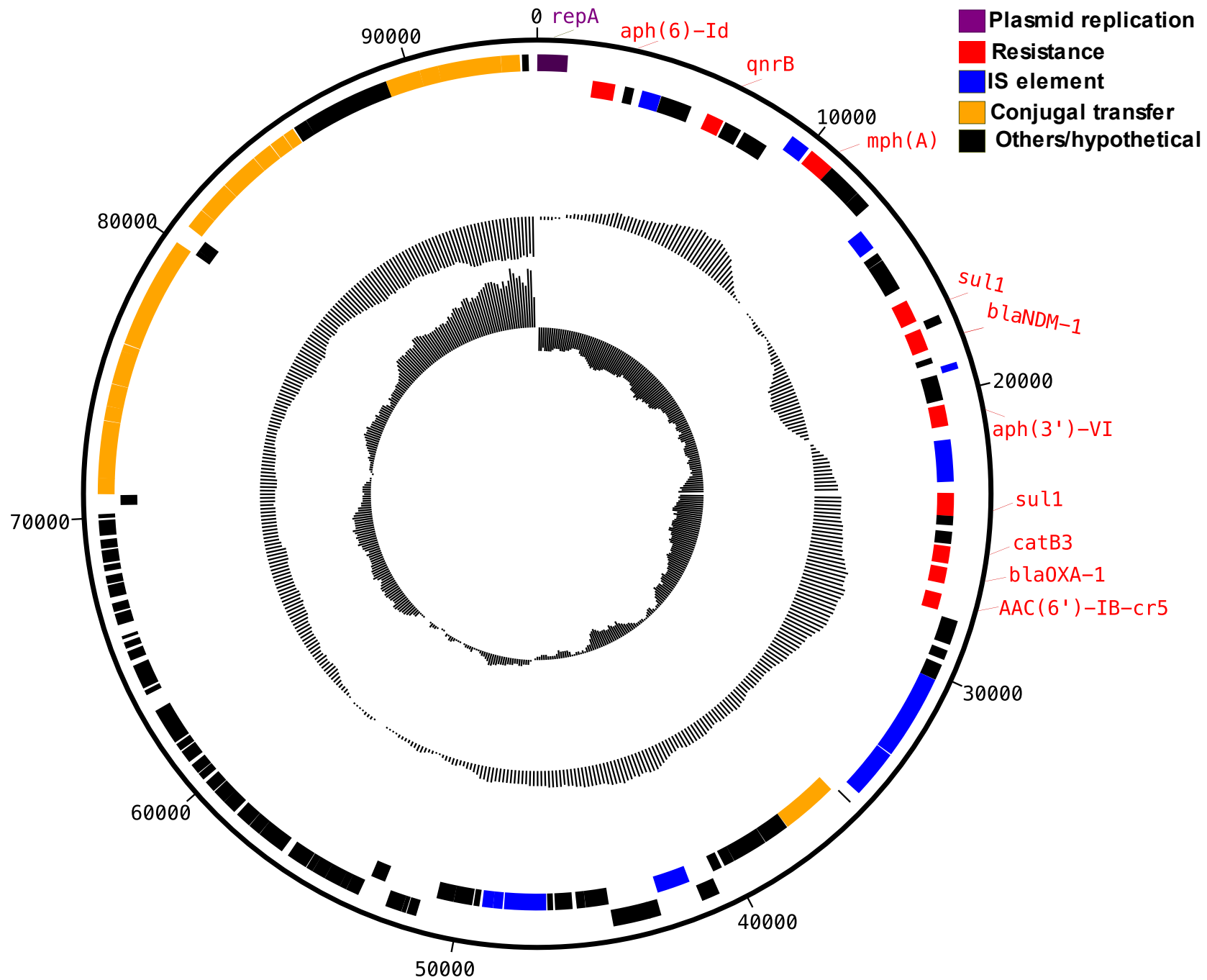

Supplement: Fig. S2 — Circular plasmid map of the novel IncL-96kb plasmid. [file msystems.01128-24-s0002.pdf]

**(A)**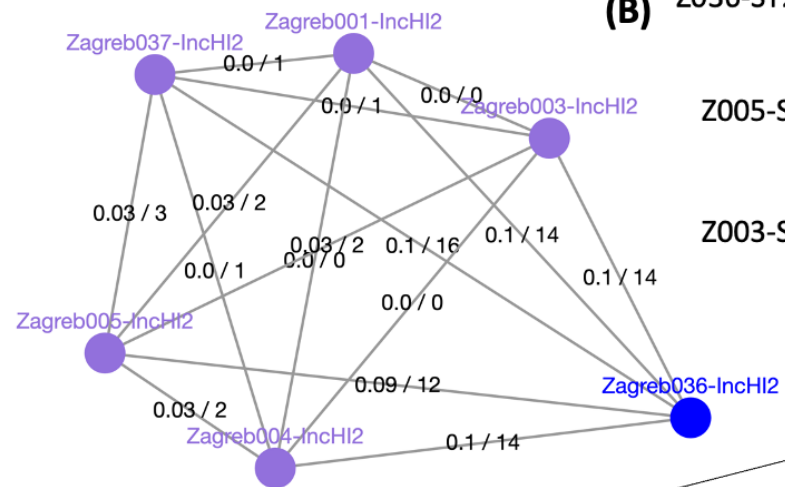**(B)**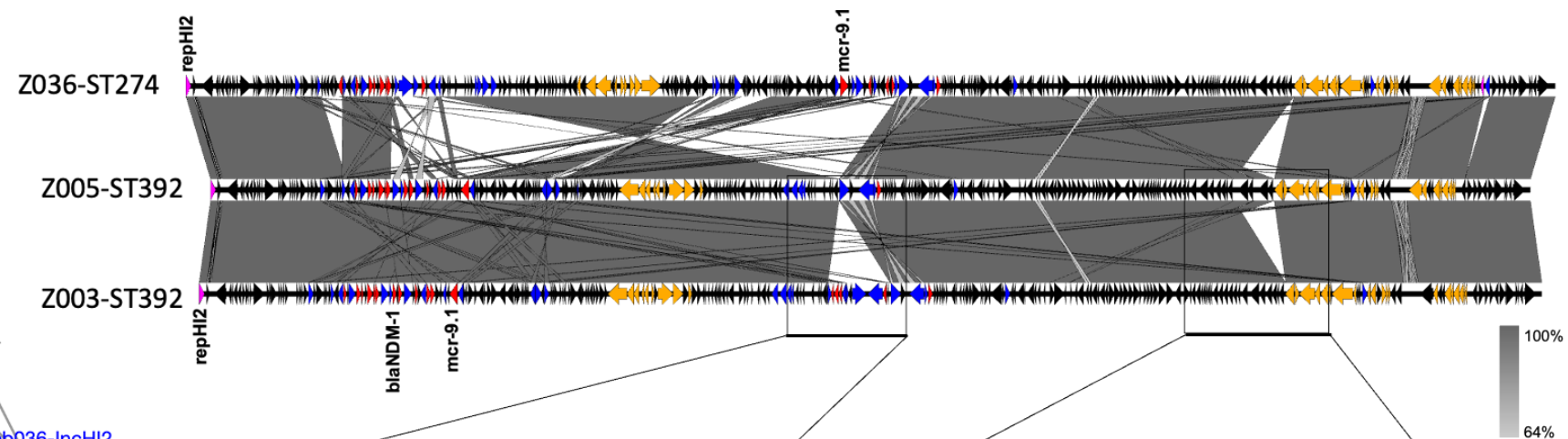**(C)**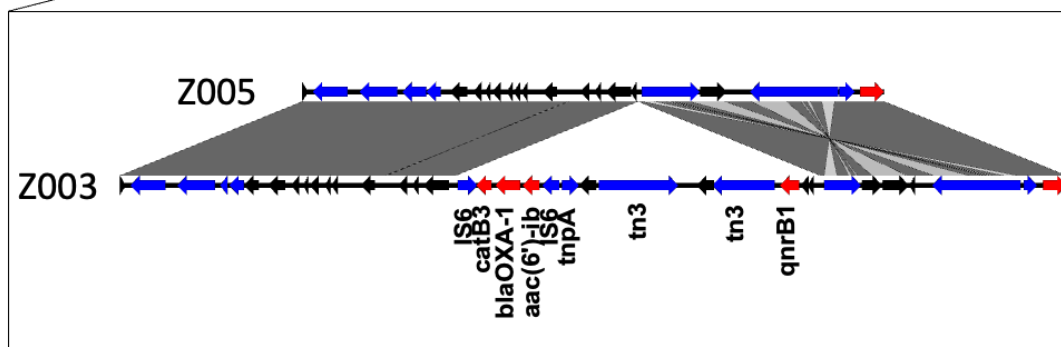**(D)**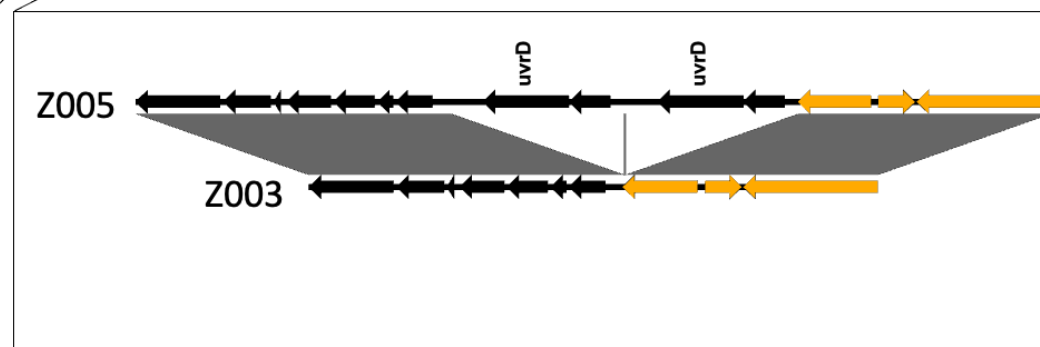

Supplement: Fig. S3 — Structural and mutational analysis of IncHI2 plasmids. [file msystems.01128-24-s0003.pdf]

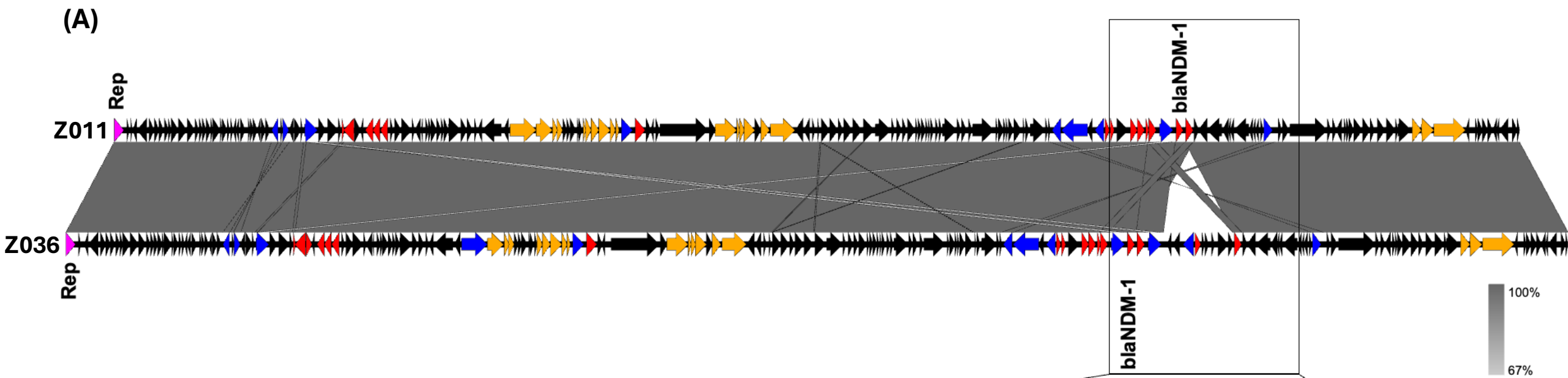

(B)

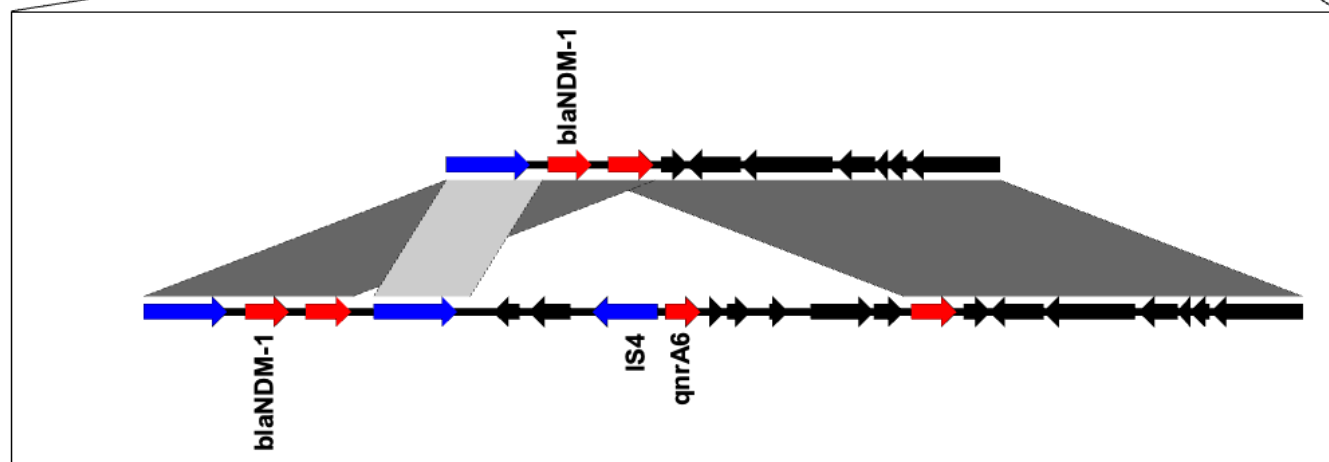

Supplement: Fig. S4 — Comparative genomic analysis of IncC plasmid. [file msystems.01128-24-s0004.pdf]

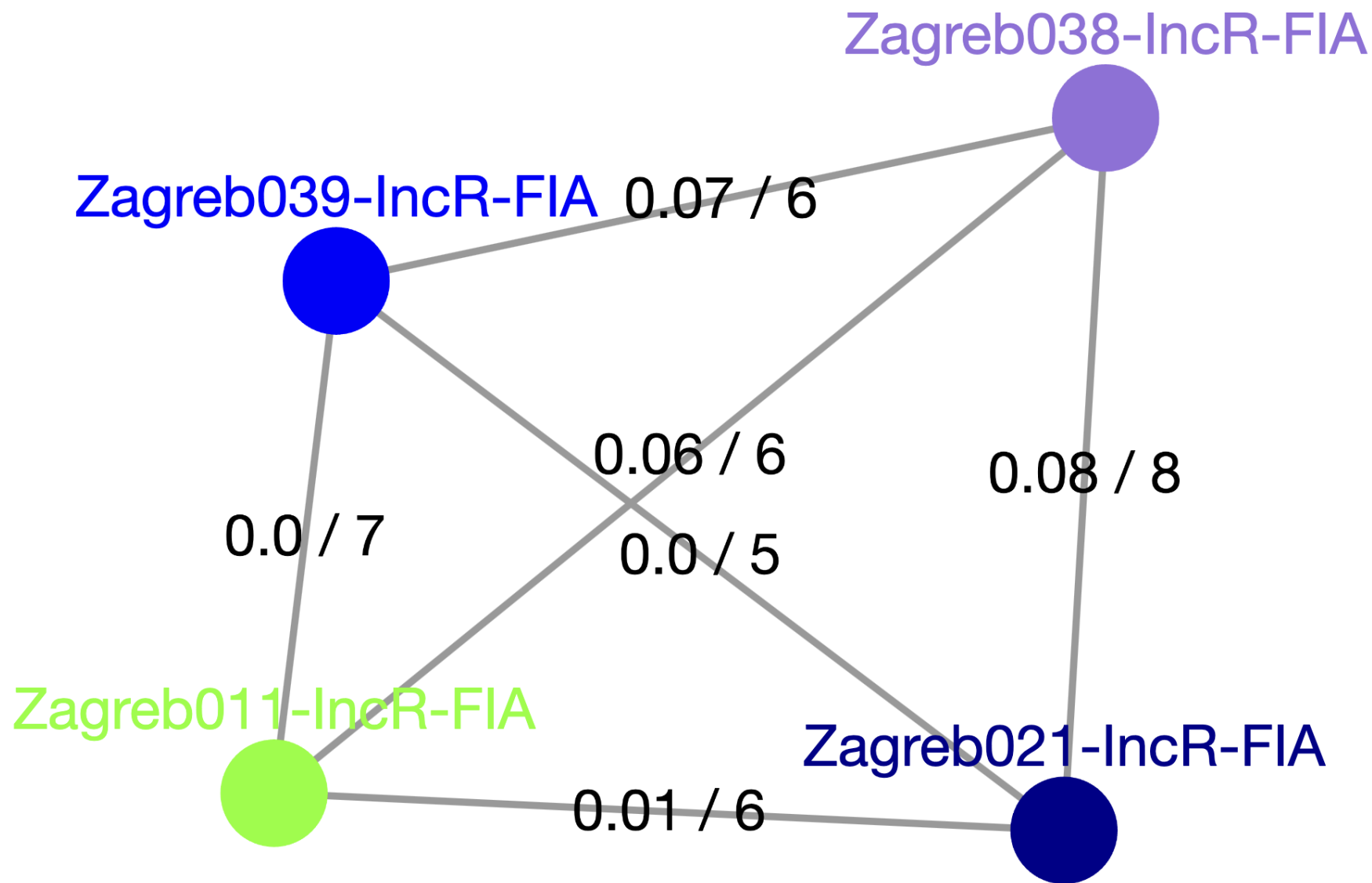

Supplement: Fig. S5 — Pling relatedness network of IncR-FIA plasmids shows differences in gene content and organization. [file msystems.01128-24-s0005.pdf]

(A)

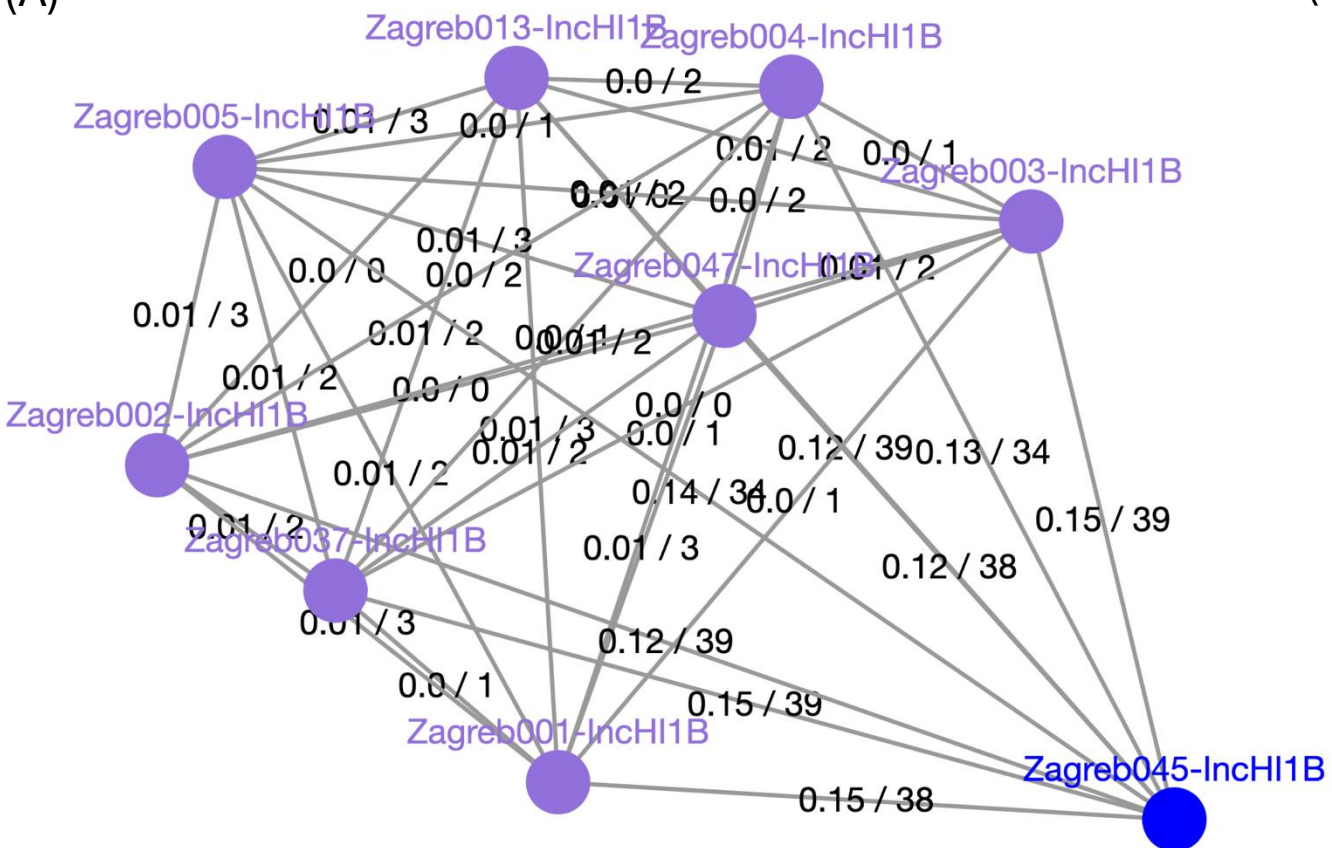

(B)

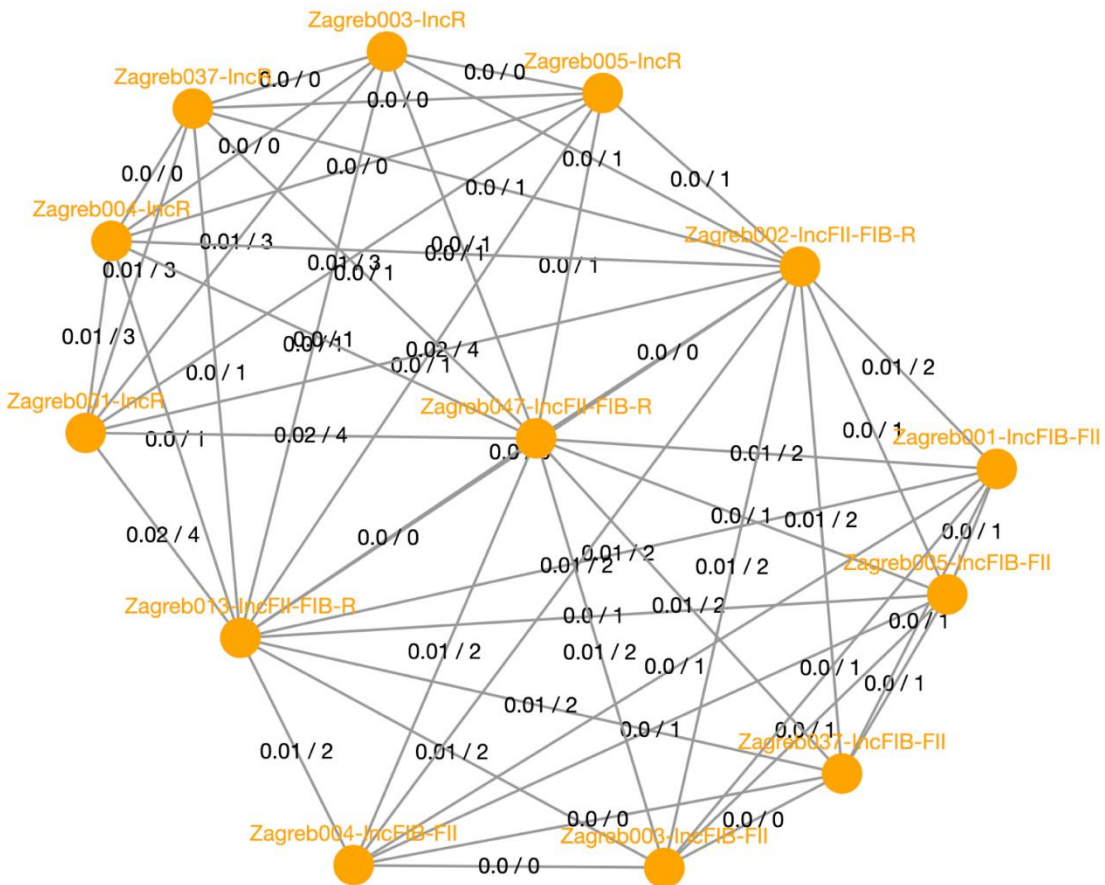

Supplement: Fig. S6 — Structural comparative genomics of the IncHI1B and IncFIB-FII-R plasmids not carrying carbapenemases. [file msystems.01128-24-s0006.pdf]
