# Supplementary material for: Tracking clonal and plasmid transmission in colistin- and carbapenem-resistant Klebsiella pneumoniae
Source: mSystems. 2025 Jan 10;10(2):e01128-24. doi: 10.1128/msystems.01128-24 (PMC11834398; doi:10.1128/msystems.01128-24)
Supplement: Legends — for supplemental material. [file msystems.01128-24-s0007.docx]

**Supplementary Material for**

**TRACKING CLONAL AND PLASMID TRANSMISSION IN COLISTIN AND CARBAPENEM RESISTANT *KLEBSIELLA PNEUMONIAE***

**Ifeoluwa Akintayo,^1^ Marko Siroglavic,^2^ Daria Frolova,^3^ Mabel Budia Silva,^1^ Hajo Grundmann,^1^ Zamin Iqbal,^3,4^ Ana Budimir,^2^ Sandra Reuter,^1^***

1Institute for Infection Prevention and Control, Faculty of Medicine, University of

Freiburg, Freiburg, Germany.

2Department of Clinical Microbiology, Infection Prevention and Control, University

Hospital Centre Zagreb, Zagreb, Croatia.

3European Molecular Biology Laboratory - European Bioinformatics Institute, Hinxton, UK.

4Milner Centre for Evolution, University of Bath, Bath, UK.

*Address correspondence to Sandra Reuter; [Sandra.reuter@uniklinik-freiburg.de](mailto:Sandra.reuter@uniklinik-freiburg.de)

**Supplementary Tables**

**Supplementary Table S1: Metadata of collection.** Information on clinical metadata, clustering and sequencing statistics, phenotypic characteristics, identified antibiotic resistance genes and mutations, as well as detected plasmid incompatibility groups.

**Supplementary Table S2:** **Overview of all detected plasmids, their incompatibility groups, sizes and colistin and/or carbapenem resistance (COL-CR) genes detected.** Same colour in a column indicates plasmid similarities according to mash tree distance.

**Supplementary Figures**

**Supplementary Figure 1: Spatial and temporal relationship of all patients within the hospital.**

**Supplementary Figure 2: Circular plasmid map of the novel IncL-_96kb_ plasmid.** The tracks from the outside to inside represent: (1) Forward Coding Sequence; (2) Reverse Coding Sequence; (3) %GC plot (4) GC skew. Genes are coloured according to functional group.

**Supplementary Figure 3: Structural and mutational analysis of IncHI2 plasmids. (A)** Structural relationship among the plasmids analysed using the pling tool, represented as a network. Each plasmid is a node. Edges are labelled with two numbers: the “containment distance” (the proportion of the smaller plasmid which is not alignable to the larger - a low distance means almost all of the small plasmid is contained in the larger) and the “double-cut and join indel distance” (the number of structural rearrangement events, including indels, separating the two genomes).   **(B)** Linear comparison of representative of ST392-cluster II isolates and ST274 (Z036). **(C)** Detailed view showing the region not found in Z005 IncHI2 plasmid, and **(D)** showing the region not found in Z003 IncHI2 plasmid. The grey area indicates regions of shared similarities. Red arrows indicate resistance genes, blue arrow indicates transposons, orange arrows indicate the conjugal transfer genes and black arrows are other genes like hypothetical or unknown genes. rep: replication system.

**Supplementary Figure 4: Comparative genomic analysis of IncC plasmid.** (A) Linear comparison of Z011 (ST101) and Z036 (ST274) and (B) detailed view showing the region not found in Z036 isolates which comprises many hypothetical proteins.

**Supplementary Figure 5: Pling relatedness network of IncR-FIA plasmids shows differences in gene content and organisation.** Apart from Z038, other plasmids had 99-100% shared sequence content but are separated by 5-7 rearrangement events.

**Supplementary Figure 6: Structural comparative genomics of the IncHI1B and IncFIB-FII-R plasmids not carrying carbapenemases. (A)** All IncHI1B plasmids (purple) except Z045 (blue) have a containment distance of 0 to 0.01, meaning that they are >99% alignable, with very little gene gain/loss. In addition, the plasmids are very similar in terms of synteny, with 1 to 3 rearrangement events separating them. **(B)** IncR, IncFIB-FII, and IncFIB-FII-R fusion plasmids. IncR plasmids (highlighted in blue) as well as IncFIB-FII plasmids (red) are very similar within their groups, with little to no rearrangements. IncFIB-FII-R plasmids (purple) are contained within both groups as indicated by the low containment distances (98100% shared sequence) and separated by 0-4 rearrangement events. This indicates a fusion of these two separate plasmids, Z002 (ST392 singleton), Z047 and Z013 (both ST392 cluster I). As these isolates come temporally later than the others (all of ST392 cluster II), we assume a fusion event to be most likely as opposed to a split into two plasmids.
